# Potential Suppressive Effects of Two C_60_ Fullerene Derivatives on Acquired Immunity

**DOI:** 10.1186/s11671-016-1663-7

**Published:** 2016-10-06

**Authors:** Toshiro Hirai, Yasuo Yoshioka, Asako Udaka, Eiichiro Uemura, Tomoyuki Ohe, Hisae Aoshima, Jian-Qing Gao, Ken Kokubo, Takumi Oshima, Kazuya Nagano, Kazuma Higashisaka, Tadahiko Mashino, Yasuo Tsutsumi

**Affiliations:** 1 Laboratory of Toxicology and Safety Science, Graduate School of Pharmaceutical Sciences, Osaka University, 1-6 Yamadaoka, Suita, Osaka 565-0871 Japan; 2 Vaccine Creation Project, BIKEN Innovative Vaccine Research Alliance Laboratories, Research Institute for Microbial Diseases, Osaka University, 3-1 Yamadaoka, Suita, Osaka 565-0871 Japan; 3 BIKEN Center for Innovative Vaccine Research and Development, The Research Foundation for Microbial Diseases of Osaka University, 3-1 Yamadaoka, Suita, Osaka 565-0871 Japan; 4 Bioorganic and Medicinal Chemistry, Faculty of Pharmacy, Keio University, 1-5-30 Shibakoen, Minato-ku, Tokyo 105-8512 Japan; 5 Vitamin C60 BioResearch Corporation, 1-3-19 Yaesu, Chuo-ku Tokyo, 103-0028 Japan; 6 Institute of Pharmaceutics, College of Pharmaceutical Sciences, Zhejiang University, Hangzhou, 310058 Zhejiang People’s Republic of China; 7 Department of Applied Chemistry, Graduate School of Engineering, Osaka University, 2-1 Yamadaoka, Suita, Osaka 565-0871 Japan; 8 Laboratory of Biopharmaceutical Research, National Institutes of Biomedical Innovation, Health and Nutrition, 7-6-8 Saitoasagi, Ibaraki, Osaka 567-0085 Japan; 9 The Center for Advanced Medical Engineering and Informatics, Osaka University, 1-6 Yamadaoka, Suita, Osaka 565-0871 Japan

**Keywords:** Acquired immunity, B cell, C_60_, Fullerene, Nanomaterial, T cell

## Abstract

The therapeutic effects of fullerene derivatives on many models of inflammatory disease have been demonstrated. The anti-inflammatory mechanisms of these nanoparticles remain to be elucidated, though their beneficial roles in allergy and autoimmune diseases suggest their suppressive potential in acquired immunity. Here, we evaluated the effects of C_60_ pyrrolidine tris-acid (C_60_-P) and polyhydroxylated fullerene (C_60_(OH)_36_) on the acquired immune response in vitro and in vivo. In vitro, both C_60_ derivatives had dose-dependent suppressive effects on T cell receptor-mediated activation of T cells and antibody production by B cells under anti-CD40/IL-4 stimulation, similar to the actions of the antioxidant *N*-acetylcysteine. In addition, C_60_-P suppressed ovalbumin-specific antibody production and ovalbumin-specific T cell responses in vivo, although T cell-independent antibodies responses were not affected by C_60_-P. Together, our data suggest that fullerene derivatives can suppress acquired immune responses that require T cells.

## Background

Fullerenes C_60_ and C_70_ are unique spherical carbon molecules. Fullerenes have a highly unsaturated structure and excellent electron-receptor properties, and for these reasons, these molecules—particularly water-soluble fullerene derivatives in which hydrophilic group moieties are added to the carbon cage for biological uses—have been investigated extensively as radical scavengers [1]. Fullerene derivatives have many beneficial biological effects supposedly related to their antioxidant activity, including liver protection [2], reduction of neuronal injury [3, 4], extension of life span [5], and UV- and radioprotection [6]. In addition, fullerenes have many potential biological roles beyond their antioxidant activity, including anti-HIV activity [7, 8] and enzyme inhibition [9].

Many studies have shown the anti-inflammatory effects of fullerene derivatives, which are expected to be candidates for use as anti-inflammatories [10–12]. However, despite the abundant evidence of these anti-inflammatory effects, the mechanisms by which they occur are not fully understood. The therapeutic effects of fullerene derivatives on allergy [13] and on autoimmune diseases [4, 14] suggest their suppressive potential in acquired immunity, but little is known about the effects of these derivatives on acquired immunity. Here, we explored the effects of fullerene derivatives on the acquired immune response in vitro and in vivo by using two hydrophilized C_60_ derivatives, namely C_60_ pyrrolidine tris-acid (C_60_-P) and polyhydroxylated fullerene (C_60_(OH)_36_). Our data suggest that fullerene derivatives can suppress acquired immune responses that require T cells and support their potential for use in the treatment of inflammatory diseases.

## Methods

### C_60_ Fullerene Derivatives

C_60_ pyrrolidine tris-acid (C_66_O_6_NH_7_) (C_60_-P) was purchased from FLOX (Kanagawa, Japan). Polyhydroxylated fullerene (C_60_(OH)_36_·8H_2_O; C_60_(OH)_36_) was synthesized as previously described [15]. We confirmed the purity (95.3 %) of C_60_-P by using liquid chromatography–mass spectrometry. C_60_(OH)_36_ is a mixture of isomers; we prepared and purified it by using a method described in the literature [15]. The C_60_ powders were stored in the dark at room temperature. Immediately before use, the powder was dispersed in dimethyl sulfoxide (Wako, Osaka, Japan), sonicated at 400 W for 5 min at 25 °C, and then vortexed for 1 min. This suspension was then diluted with cell culture medium or saline, further sonicated at 400 W for 5 min at 25 °C, and then vortexed for 1 min.

### Reagents

Mitomycin C was purchased from Wako. Anti-mouse CD3ε (145-2C11), CD28 (37.51), and CD40 (HM40-3) were obtained from Biolegend (San Diego, CA, USA). Recombinant mouse IL-4 was purchased from R&D Systems (Minneapolis, MN, USA). Ovalbumin (OVA), *N*-acetylcysteine (NAC), and lipopolysaccharide (LPS; *Escherichia coli O55:B5*) were purchased from Sigma–Aldrich (St. Louis, MO, USA). NP_49_-AECM-Ficoll (NP_49_-Ficoll; 4-hydroxy-3-nitrophenylacetic hapten conjugated to amino-ethyl-carboxy-methyl-Ficoll) and NP_30_-BSA were obtained from Biosearch Technologies (Novato, CA, USA).

### Mice

Female C57BL/6 and BALB/c mice were purchased from SLC (Kyoto, Japan) and used at 6 to 8 weeks of age.

### Mixed Lymphocyte Reactions (MLRs)

Responder splenocytes from BALB/c mice were seeded onto 96-well flat-bottomed plates (Nunc, Roskilde, Denmark) (1 × 10^6^ cells/well). Stimulator splenocytes from C57BL/6 mice were treated with 250 μg/mL mitomycin C at 37 °C for 30 min, followed by three extensive washes with complete RPMI1640 supplemented with 10 % fetal bovine serum, 10 mL/L of a 100× nonessential amino acid solution (Gibco, Invitrogen, Carlsbad, CA, USA), 50 μM 2-mercaptoethanol (Gibco), and 1 % antibiotic cocktail (10,000 U/mL penicillin, 10,000 μg/mL streptomycin, 25 μg/mL amphotericin B; Gibco). They were then added at 3 × 10^6^ cells/well to responder splenocytes. C_60_-P or C_60_(OH)_36_ was added to responder splenocytes 30 min before addition of the stimulator splenocytes. After incubation of the cells for 4 days at 37 °C (95 % room air, 5 % CO_2_), the amount of interleukin 2 (IL-2) released into an aliquot of culture supernatant was measured with a murine IL-2 enzyme-linked immunosorbent assay (ELISA) kit (eBioscience, San Diego, CA, USA) in accordance with the manufacturer’s instructions.

### OVA-Specific Immune Response In Vitro

C57BL/6 mice were intraperitoneally immunized with OVA (10 μg/mouse) and Imject Alum adjuvant (1 mg/mouse) (Thermo Fisher Scientific K.K., Tokyo, Japan) weekly. One week after the third injection, the mice were euthanized, and single-cell suspensions of splenocytes were prepared. C_60_-P or C_60_(OH)_36_ was added to the well 30 min before the addition of OVA (100 μg/mL). After incubation of the cells for 3 days at 37 °C (95 % room air, 5 % CO_2_), the amount of interleukin-4 (IL-4) released into an aliquot of culture supernatant was measured with an IL-4 ELISA kit (eBioscience) in accordance with the manufacturer’s instructions.

### T cell stimulation assay

To stimulate CD4^+^ T cells, we coated 96-well flat-bottomed plates (Nunc) with anti-CD3ε (2.5 μg/mL) by overnight incubation at 4 °C. CD4^+^ T cells were negatively isolated with a CD4^+^ T cell isolation kit (Miltenyi Biotec, Bergisch-Gladbach, Germany) in accordance with the manufacturer’s instructions. CD4^+^ T cell purity was determined by flow cytometry (CD3^+^, CD4^+^ cells >90 % purification). Immediately after cell preparation, CD4^+^ T cells were added to the coating plate (1 × 10^5^ cells/well). C_60_-P, C_60_(OH)_36_, or NAC was added to the well 30 min before the addition of anti-CD28 (1 μg/mL). After incubation of the cells for 3 days at 37 °C (95 % room air, 5 % CO_2_), the amount of IL-2 released into an aliquot of culture supernatant was measured with an IL-2 ELISA kit (eBioscience) in accordance with the manufacturer’s instructions.

### Class-Switch Assays

B cells were purified from splenocytes by positive selection with CD19 MicroBeads (Miltenyi Biotec) in accordance with the manufacturer’s instructions. B cell purity was determined by flow cytometry (B220^+^ cells >90 % purification). Immediately after cell preparation, B cells were seeded into 96-well flat-bottom plates (Nunc) (1 × 10^6^ cells/well). B cells were incubated in complete RPMI1640 with 10 ng/mL IL-4 and 3 μg/mL anti-CD40 for 10 days. C_60_-P, C_60_(OH)_36_, or NAC was added to the wells 30 min before the addition of IL-4 and anti-CD40. After incubation of the cells for 10 days at 37 °C (95 % room air, 5 % CO_2_), the amount of total IgE released into an aliquot of culture supernatant was measured with a total IgE ELISA kit (BD Biosciences) in accordance with the manufacturer’s instructions.

### In Vivo Treatment with a Mixture of C_60_-P and Antigen

C57BL/6 mice were treated weekly with OVA (10 μg/mouse), LPS (30 μg/mouse), or NP_49_-Ficoll (50 μg/mouse), or with a mixture of one of these plus C_60_-P (62.5 to 250 μg/kg), by intraperitoneal injection. Seven days after the third (in the case of the OVA experiment) or second (in the case of the LPS and NP-Ficoll experiments) treatments, the blood or blood and spleens, were collected to evaluate antibody responses and effector cytokine responses. Splenocytes (1 × 10^6^ cells/well) were re-stimulated with OVA (100 μg mL). After incubation of the cells for 72 h at 37 °C, the levels of IL-4 released into an aliquot of culture supernatant were measured by ELISA (eBioscience) in accordance with the manufacturer’s instructions.

### Detection of Antigen-Specific Antibodies

Plasma levels of antigen-specific antibodies were determined by ELISA. To detect OVA-specific IgG1, LPS, or NP-specific IgM or IgG3, we coated ELISA plates (Maxisorp; Nunc) with OVA (10 μg/mL), LPS (25 μg/mL), or NP_30_-BSA (5 μg/mL). The coated plates were incubated with 2 % Block Ace (for OVA-coated plates) (Dainippon Sumitomo Pharmaceuticals, Osaka, Japan) or 1 % BSA (for LPS or NP_30_-BSA-coated plates) for 2 h at room temperature. Plasma dilutions were added to the antigen-coated plates. After incubation with the plasma for 2 h at room temperature, the coated plates were incubated with a horseradish peroxidase-conjugated goat anti-mouse IgG1, IgM, or IgG3 solution (SouthernBiotech, Birmingham, AL, USA) for 2 h at room temperature. After the incubation, the color reaction was developed with tetramethylbenzidine (Moss, Inc.; Pasadena, MD, USA), stopped with 2 N H_2_SO_4_, and measured at OD_450–620_ on a microplate reader. To detect OVA-specific IgE, we coated ELISA plates with purified rat anti-mouse IgE (2 μg/mL) and detected OVA-specific IgE with biotin-conjugated OVA (5 μg/mL) followed by horseradish peroxidase-coupled streptavidin (Southern Biotechnology Associates, Birmingham, AL).

### Statistical Analysis

Statistical analyses were performed with Ekuseru-Toukei 2012 software (Social Survey Research Information Co., Ltd., Tokyo, Japan). Significant differences between control groups and C_60_-P-added groups were determined by using the Williams test; a *P* value less than 0.05 was considered significant.

## Results

### Effects of C_60_ Derivatives on T cells In Vitro

We used two hydrophilized C_60_ derivatives, namely C_60_-P and C_60_(OH)_36_, which had shown the strongest anti-inflammatory effects among more than 20 C_60_ derivatives that we had screened by using IL-1β-stimulated Caco-2 cells (human colon epithelial carcinoma cells) (paper in preparation). In a preliminary experiment, to evaluate the immune suppressive effects of C_60_-P and C_60_(OH)_36_, we evaluated the cytotoxicity of the C_60_ derivatives on murine splenocytes by using a lactate dehydrogenase assay. After 3 days’ co-incubation with each C_60_ derivative, no cytotoxicity was induced by either one, at least at the maximum dose that we used, 100 μM (data not shown). We therefore used 100 μM of each C_60_ derivative as the maximum dose in the following in vitro assays.

**Fig. 1:**
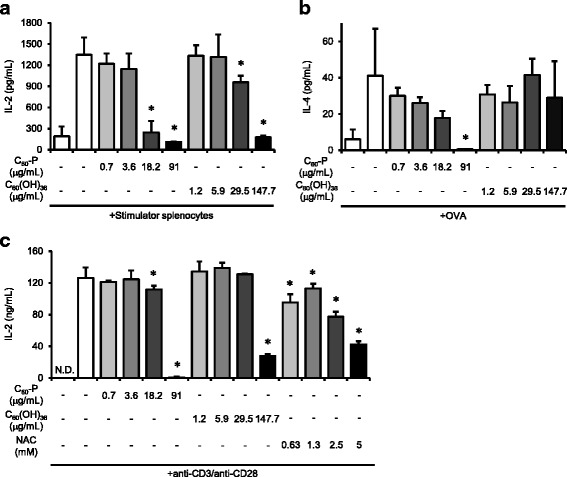
Effects of C_60_ derivatives on T cell responses. **a** C_60_-P or C_60_(OH)_36_ was added to responder splenocytes 30 min before the addition of stimulator splenocytes. After incubation of the cells for 4 days, the amount of IL-2 in the supernatants was measured by ELISA. **b** One week after the third immunization with OVA and alum, mice were euthanized and single-cell suspensions of splenocytes were prepared. C_60_-P or C_60_(OH)_36_ was added to the wells 30 min before the addition of OVA (100 μg/mL). After incubation of the cells for 3 days, the amount of IL-4 in the supernatants was measured by ELISA. **c** Purified CD4^+^ T cells were added to anti-CD3-coated plates. Each C_60_ derivative or *N*-acetylcysteine (NAC) was added to the wells 30 min before the addition of anti-CD28. After incubation of the cells for 3 days, the amount of IL-2 in the supernatants was determined by ELISA. Data are means ± SDs for three to six independent cultures (*n* = 3 to 6). *P < 0.05 vs. control group

The MLR is one of the assays most commonly used to evaluate the T cell-mediated immune suppressive effects of chemicals and is essentially based on the alloantigen-specific T cell immune response. We first evaluated the effect of C_60_ derivatives on T cells by using MLR and measured IL-2 as a mediator of T cell expansion. C_60_-P and C_60_(OH)_36_ dose-dependently suppressed IL-2 production by responder cells (Fig. 1a). Thus, both C_60_ derivatives had some T cell-suppressive effect. Next, each C_60_ derivative was added to splenocytes with OVA in a model of antigen-immunized splenocyte culture to evaluate the effects of the derivatives on the OVA-specific T cell immune response. IL-4 production induced by OVA-re-stimulated splenocytes was significantly reduced by C_60_-P treatment (Fig. 1b), but not by C_60_(OH)_36_. To confirm the effect of C_60_-P on T cells, CD4^+^ T cells were purified from splenocytes and then stimulated with anti-CD3 and anti-CD28. To some groups of CD4^+^ T cells, we added each C_60_ derivative or the antioxidant NAC at various concentrations 30 min before the addition of anti-CD28 antibodies. IL-2 production induced by anti-CD3 and anti-CD28 stimulation was decreased by both C_60_ derivatives (Fig. 1c). We concluded that the C_60_ derivatives had T cell-suppressive effects in vitro at least. In addition, NAC suppressed IL-2 production, suggesting that the effects of C_60_ derivatives on T cells were related to their antioxidant properties.

### Effects of C_60_ Derivatives on B cells In Vitro

**Fig. 2:**
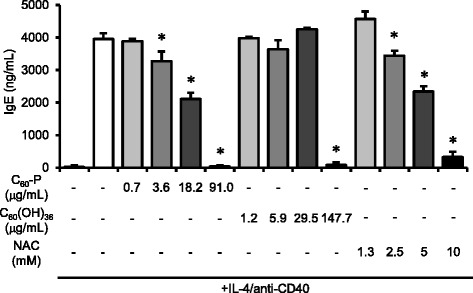
Effects of C_60_ derivatives on B cell responses. C_60_-P, C_60_(OH)_36_, or *N*-acetylcysteine (NAC) was added to purified B cells 30 min before the addition of IL-4 and anti-CD40. After incubation of the cells for 10 days, the amount of total IgE in the supernatants was measured by ELISA. Data are means ± SDs for three to six independent cultures (*n* = 3 to 6). *P < 0.05 vs. control group

We evaluated the effects of the C_60_ derivatives on antibody production by B cells in vitro by using a class-switch assay. Briefly, CD19^+^ B cells were purified from splenocytes and incubated with IL-4 and anti-CD40 for 10 days to provoke their differentiation into IgE-producing plasma cells. We added one of the C_60_ derivatives or NAC at various concentrations 30 min before adding the IL-4 and anti-CD40. We evaluated the effects of both C_60_ derivatives on antibody production by measuring IgE levels in the culture supernatant on day 10. Both C_60_ derivatives dose-dependently suppressed IgE production (Fig. 2); the effects of C_60_-P appeared more potent than those of C_60_(OH)_36_. Our results suggested that the C_60_ derivatives had some suppressive effects on B cells. In addition, NAC treatment significantly decreased IgE levels, suggesting that the decrease in IgE production caused by the C_60_ derivatives is associated with their antioxidant properties.

### Effects of C_60_-P on the Acquired Immune Response In Vivo

**Fig. 3:**
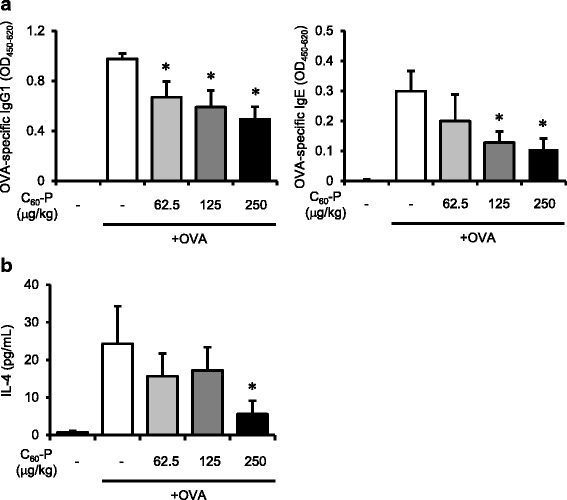
Effects of C_60_-P on acquired immune response to ovalbumin (OVA) in vivo. C57BL/6 mice were treated weekly with OVA alone (10 μg/mouse) or with a mixture of C_60_-P at varying dose rates and OVA by intraperitoneal injection. Seven days after the last treatment, spleens and plasma were collected. **a** Levels of OVA-specific IgG1 and IgE in the plasma were evaluated by ELISA. **b** Splenocytes were re-stimulated with OVA (100 μg mL). After incubation of the cells for 72 h, levels of IL-4 in the supernatants were measured by ELISA. Data are means ± SEMs (*n* = 4 or 5). *P < 0.05 vs. OVA alone group

Our in vitro evaluation of the effects of the C_60_ derivatives revealed that both had the potential to suppress T and B cells; overall, C_60_-P seemed to have more potent effects than C_60_(OH)_36_ (Figs. 1 and 2). In the next part of our experiment, we therefore used C_60_-P to evaluate the effects on the acquired immune response to OVA in vivo*.* Mice were treated either with OVA alone or with C_60_-P plus OVA every week for 3 weeks by intraperitoneal injection. One week after the last treatment, we evaluated OVA-specific antibody production and the T cell immune response. OVA treatment alone induced OVA-specific IgG1 and IgE production (Fig. 3a). Both OVA-specific IgG1 and IgE levels were decreased by co-administration of C_60_-P in a dose-dependent manner. In addition, IL-4 production by OVA-re-stimulated splenocytes from OVA + C_60_-P-treated mice was significantly lower (when 250 μg/kg was used) than that in mice treated with OVA alone (Fig. 3b). These findings showed that C_60_-P could exert suppressive effects on the acquired immune response in vivo.

**Fig. 4:**
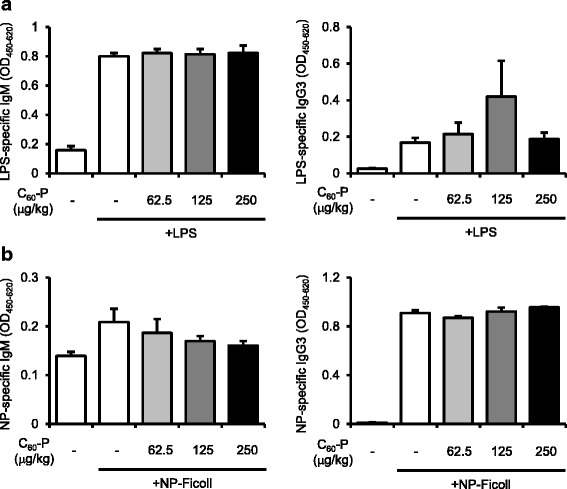
Effects of C_60_-P on T cell-independent antibody responses in vivo. C57BL/6 mice were treated weekly with lipopolysaccharide (LPS) (30 μg/mouse) or NP_49_-Ficoll (50 μg/mouse) alone or with a mixture of C_60_-P at varying dose rates and LPS or NP_49_-Ficoll by intraperitoneal injection. Seven days after the second treatment, plasma was collected. Levels of LPS-specific (**a**) or NP-specific (**b**) IgM and IgG3 were evaluated by ELISA. Data are means ± SEMs (*n* = 5). *P < 0.05 vs. LPS or NP_49_-Ficoll alone group

Antibody production is classified as T dependent or T independent on the basis of the requirement for T cell help in antibody production [16]. T-dependent antigens are proteins such as OVA, and production of OVA-specific antibodies needs the cognate help of OVA-specific T cells. In contrast, the T-independent antibody response requires only B cell activation. To gather mechanistic information on the in vivo effects of C_60_-P—particularly in regard to whether they were solely B cell effects—we evaluated the effects of C_60_-P on T cell-independent antibody production. Because T-independent antigens are categorized into types I and II according to their B cell-activating mechanisms, we used LPS as the type I antigen and NP-Ficoll as the type II antigen. Mice were immunized with either LPS or NP_49_-Ficoll alone or with either of these plus C_60_-P weekly for 2 weeks. One week after the last immunization, we evaluated the levels of LPS- or NP-specific IgM and IgG3 as T-independent antibodies. Levels of LPS-specific IgM or IgG3 induced by LPS injection were not affected by co-injection of C_60_-P (Fig. 4a). Similarly, levels of NP-specific IgM or IgG3 induced by NP_49_-Ficoll were not significantly changed by C_60_-P (Fig. 4b). Thus, the in vivo suppressive effect of C_60_-P on the acquired immune response could not be explained by a simple direct inhibitory effect on B cell activation.

## Discussion

According to our in vitro results, our C_60_ derivatives appeared to have a kind of direct suppressive effect on T cell and B cell activation (Figs. 1 and 2), and C_60_-P co-administered with OVA suppressed the OVA-specific immune response in vivo. Both derivatives had suppressive effects on T and B cells, suggesting that the suppressive effects on acquired immunity might not be confined to particular fullerene derivatives but might be general properties related to the special structure of these molecules.

Reactive oxygen species (ROS) are important messengers in TCR signaling [17, 18]; suppression of the signaling role of ROS is consistent with the suppressive effect of NAC on T cells (Fig. 1c). The strong antioxidant properties of fullerene derivatives may enable them to work as antioxidants for T cells, suppressing TCR signal-related activation and proliferation (Fig. 1). In addition, both of our C_60_ derivatives and NAC suppressed IgE production by B cells (Fig. 2). B cell activating signaling via CD40 is dependent on ROS production [19]. Thus, B cell activation and class-switching may depend on ROS, and our C_60_ derivatives therefore suppressed B cells via their antioxidant properties. To summarize, the in vitro effects of the derivatives on both T cells and B cells are likely due to their antioxidant properties.

In contrast, in vivo, C_60_-P successfully suppressed OVA-specific immune responses, which require T cells (Fig. 3), but it did not suppress T cell-independent antibody production (Fig. 4). CD40 ligation on B cells, which is provided by T cells, is not needed for T cell-independent antibody production. Thus, the in vitro results for B cells cannot be extrapolated directly to the results regarding T cell-independent antibody production in vivo*.* These findings would be in conflict if the in vivo suppressive effect on the OVA-specific immune response were induced by the targeting of B cells or by the antioxidant properties of the derivatives alone. C_60_-P likely exists as aggregates or agglomerates of varying sizes (transmission electron microscopic observations by another group have revealed an average size of C_60_-P of 45.6 ± 18.8 nm; [20]). Nanoparticles of that size are probably primarily ingested by macrophages/Kupffer cells in vivo [21]; thus, direct interaction between C_60_-P and T or B cells is less likely to occur in our in vivo experimental system than in our in vitro system. We therefore speculate that the pharmacokinetics of C_60_-P were responsible for the discrepancy between its in vivo and in vitro effects. Further studies are needed to conclusively identify the cellular and molecular mechanisms of the effects of C_60_-P in vivo.

The acquired immune response is essentially the result of complex interactions among T cells, B cells, and dendritic cells (DCs). C_60_ and C_60_-P have been suggested to promote antigen presentation by DCs by another group [20]. C_60_(OH)_36_, which might have weaker suppressive effects than C_60_-P on T and B cells, failed to suppress OVA-specific IL-4 production by OVA-immunized splenocytes (Fig. 1b). In that assay, antigen-presentation by the DC and macrophage components of splenocytes played a critical role in inducing OVA-specific IL-4 production. Thus, the effects of C_60_(OH)_36_ on T cells and DCs might have canceled each other out. Thus, it is possible that C_60_ derivatives inconsistently affect each aspect of the acquired immune response in vivo. However, considering the ultimate phenotype of the OVA-specific immune response induced by C_60_-P in vivo appeared to be suppressive (Fig. 3), together we may say that fullerene derivatives can be suppressive reagents for acquired immune responses in vivo*.﻿*

## Conclusions

Our data suggest that fullerene derivatives can suppress acquired immune responses that require T cells. Some groups have attempted to use fullerenes as tumor-inhibitory reagents by activating anti-tumor immunity [22, 23]. With this use, the suppression of acquired immunity by the fullerene can be a side effect. Because fullerene derivatives may have variable efficacies and may have the potential to have conflicting actions in some situations like a conflict of effect on DC, T, and B cells above, future studies to elucidate the molecular mechanisms of their efficacy are needed so that we can take full advantage of their beneficial effects in each situation.

## Abbreviations

C_60_(OH)_36_: Polyhydroxylated fullerene (C_60_(OH)_36_·8H_2_O)
C_60_-P: C_60_ Pyrrolidine tris-acid (C_66_O_6_NH_7_)
ELISA: Enzyme-linked immunosorbent assay
IL: Interleukin
LPS: Lipopolysaccharide
NAC: *N*-acetylcysteine
OVA: Ovalbumin
TCR: T cell receptor
